# Durability of Tricuspid Valve Repair in Patients Undergoing Left Ventricular Assist Device Implantation

**DOI:** 10.3390/jcm13051411

**Published:** 2024-02-29

**Authors:** Gloria Färber, Imke Schwan, Hristo Kirov, Marcel Rose, Sophie Tkebuchava, Ulrich Schneider, Tulio Caldonazo, Mahmoud Diab, Torsten Doenst

**Affiliations:** 1 Department of Thoracic and Cardiovascular Surgery, Saarland University Medical Center, 66421 Homburg/Saar, Germany; 2 Department of Cardiothoracic Surgery, Jena University Hospital, Friedrich-Schiller-University Jena, 07743 Jena, Germany; 3 Department of Cardiac Surgery, Rotenburg Heart and Vascular Centre, 36199 Rotenburg an der Fulda, Germany

**Keywords:** left ventricular assist device, tricuspid regurgitation

## Abstract

**Objectives:** Benefits of tricuspid valve repair (TVR) in left ventricular assist device (LVAD) patients have been questioned. High TVR failure rates have been reported. Remaining or recurring TR was found to be a risk factor for right heart failure (RHF). Therefore, we assessed our experience. **Methods:** Since 12/2010, 195 patients have undergone LVAD implantation in our center. Almost half (n = 94, 48%) received concomitant TVR (LVAD+TVR). These patients were included in our analysis. Echocardiographic and clinical data were assessed. Median follow-up was 2.8 years (7 days–0.6 years). Results were correlated with clinical outcomes. **Results:** LVAD+TVR patients were 59.8 ± 11.4 years old (89.4% male) and 37.3% were INTERMACS level 1 and 2. Preoperative TR was moderate in 28 and severe in 66 patients. RV function was severely impaired in 61 patients reflected by TAPSE-values of 11.2 ± 2.9 mm (vs. 15.7 ± 3.8 mm in n = 33; *p* < 0.001). Risk for RHF according to EUROMACS-RHF risk score was high (>4 points) in 60 patients, intermediate (>2–4 points) in 19 and low (0–2 points) in 15. RHF occurred in four patients (4.3%). Mean duration of echocardiographic follow-up was 2.8 ± 2.3 years. None of the patients presented with severe and only five (5.3%) with moderate TR. The vast majority (n = 63) had mild TR, and 26 patients had no/trace TR. Survival at 1, 3 and 5 years was 77.4%, 68.1% and 55.6%, 30-day mortality was 11.7% (n = 11). Heart transplantation was performed in 12 patients (12.8%). **Conclusions:** Contrary to expectations, concomitant TVR during LVAD implantation may result in excellent repair durability, which appears to be associated with low risk for RHF.

## 1. Introduction

It is generally accepted that the degree of tricuspid valve regurgitation (TR) is directly related to increased mortality [1]. Current evidence supports the concept that elimination or reduction of TR improves symptoms and survival [2,3]. However, survival benefits of tricuspid valve repair (TVR) have been questioned as reported outcomes are inconsistent [4]. Nevertheless, TR is related to both right heart failure (RHF) and liver dysfunction [5,6].

Right heart function, in particular, is key for successful LVAD therapy. Postoperatively after LVAD implantation, the right (unsupported) heart is faced with the “hemodynamic challenge” of managing the normalized cardiac output provided by the LVAD and therefore requires special attention to prevent RHF. RHF is a common complication with reported rates of 4–50% and associated with increased morbidity and mortality [7,8,9,10,11]. RHF is still a leading cause of death, especially in the early postoperative phase [12] with a 6-month mortality up to 29% [13]. 

However, the pathophysiology of RHF in LVAD patients is not yet fully understood [14,15], but the role of the tricuspid valve is increasingly recognized and TR has been discussed as a root cause or symptom of RHF [16,17]. Irrespective of this, TR itself has been shown to be a risk factor for RHF in patients undergoing LVAD surgery [18] that may suggest performing concomitant TVR. However, additional benefits of TVR as a concomitant procedure are uncertain [19,20] and limited data exist regarding the efficacy and durability of TVR. High early postoperative TVR failure rates have been reported and remaining or recurring TR has been found to be an independent risk factor for RHF [18]. Similarly, recent studies discuss not only TR but also isolated annular dilation as a risk factor for RHF [16,17]. 

Based on the current controversies of the role of concomitant TVR in LVAD surgery, a cautious interpretation of the data may indicate that we are treating TR too late in the course of right ventricular disease and therefore are unable to stop the progress of right ventricular and end-organ dysfunction. Therefore, it had been our strategy to repair the tricuspid valve concomitantly at an early stage of TR as recommended in the current ESC/EACTS guidelines for the management of valvular heart disease (primary TR: moderate or severe TR; secondary TR: mild or moderate TR, annulus ≥40 mm or >21 mm/m^2^) [21]. Thus, we retrospectively assessed the rate of TVR and right heart failures in our cohort.

## 2. Patients and Methods

### 2.1. Ethical Statement

Our institutional review board (ethics committee, Friedrich-Schiller-Universität Jena, Jena Germany) approved the data for research and waived the need for individual informed consent (reference number: 2021-22368 and date of approval 16 June 2021).

### 2.2. Study Population

From December 2010 to March 2022, a total of 195 patients underwent LVAD implantation in our department. Of those, 94 (48%) fulfilled the criteria for TVR according to the ESC/EACTS guidelines for the management of valvular heart disease (primary TR: moderate or severe TR; secondary TR: mild or moderate TR, annulus ≥40 mm or >21 mm/m^2^) [21] and received concomitant TVR. Data had been collected prospectively and were analyzed retrospectively. 

Left ventricular assist device implantation (HVAD™—Medtronic, HeartAssist 5—Micromed Cardiovascular, HeartMate II and HeartMate 3—Abbott) was performed via a median sternotomy in all 94 patients. In all patients, outflow grafts were connected to the ascending aorta and TVR was performed using band annuloplasty on the beating heart prior to LVAD implantation. 

Perioperative and follow-up data (echocardiographic and clinical data) were assessed. TVR failure was defined as moderate or severe TR (TR > I°) at any follow-up echocardiographic examination after LVAD implantation.

All patients were followed with clinic visits three to five times per year. Median follow-up was 2.8 ± 2.3 years and ranged from 7 days to 10.6 years. Standardized blood pressure and LVAD speed adjustment were performed. We generally aim for aortic valve opening at least every three heart beats. Echocardiography prior to LVAD implantation and during follow-up included semiquantitative assessment of TR according to current guidelines [22] using color Doppler. Tricuspid annulus dimensions and determinants of RV function were measured in four-chamber view. Late right heart failure was defined as readmission with clinical indicators for RHF (i.e., ascites, peripheral edema, elevated jugular/central venous pressure) [16].

## 3. Statistics

All statistical analyses and figures were performed using SPSS 27.0 (IBM SPSS Statistics for Windows. Armonk, NY, USA: IBM Corp). Continuous variables are expressed as mean ± standard deviation, categorical data are shown as crude numbers and percentages. analysis of variance (ANOVA) with the Bonferroni algorithm was performed to compare paired variables at different times. The date of first occurrence of mild, moderate or severe TR grades was recorded for time-to-event calculation. Time-dependent calculations for survival and incidence of TR were computed by Kaplan–Meier analysis, group comparisons were made using a log-rank test with a significance level of <0.05.

## 4. Results

Table 1 shows the preoperative patient characteristics. Patients were on average 60 years old and the vast majority were male (n = 84, 89.4%). Fifty-six patients (59.6%) had undergone previous pacemaker or ICD implantation and ten (10.6%) suffered from COPD. Thirty-seven patients (39.4%) received LVAD implantation for ischemic cardiomyopathy and the rest for dilated cardiomyopathy. Thirty-five patients (37.3%) presented in a preoperative critical state defined by INTERMACS levels 1 and 2 with twelve patients (12.8%) bridged with short-term MCS. Treatment strategy was destination therapy in 40 patients (42.6%), bridge to transplantation in 31 (33%) and bridge to decision in 23 (24.5%). All these patients fulfilled the criteria for tricuspid valve repair according to the current ESC/EACTS guidelines for the management of valvular heart disease [21] with 28 patients (29.8%) presenting with moderate and 66 (70%) with severe TR. Furthermore, there was evidence for impaired RV function with an average TAPSE of 12.6 ± 3.8 mm. Risk for RHF according to EUROMACS-RHF risk score [12] was low (0–2 points) in 15 patients (15.9%), intermediate (>2–4 points) in 19 (20.2%) and high (>4 points) in 60 patients (63.8%).

Table 2 shows the operative data of the entire cohort. Average duration of surgery was 3.8 h with 111 min of cardiopulmonary bypass time and 20% redo procedures (n = 19). LVAD implantation was performed using HeartMate II in 14 patients (14.9%), HeartMate 3 in 71 (75.3%), HVAD in 6 (6.4%) or HeartAssist 5 in 3 (3.2%). For tricuspid valve annuloplasty, three different band devices were used. The majority of patients received Medtronic Simplici-T (n = 47, 50.0%), 46 patients (48.9%) received Cosgrove-Edwards and 1 (1.1%) Medtronic Simulus. Further concomitant procedures mainly consisted of aortic valve replacement (n = 14, 14.9%), closure of ASD (n = 18, 19.1%) or CABG (n = 3, 3.2%). During the postoperative course, temporary RVAD implantation was necessary in 2 patients (2.1%) and dialysis due to renal failure in 18 (19.1%) patients. In none of the patients did pump thrombosis occur, arrythmia was seen in seven (7.4%), visceral ischemia in two (2.1%) and perioperative stroke in five (5.3%) patients.

**Figure 1:**
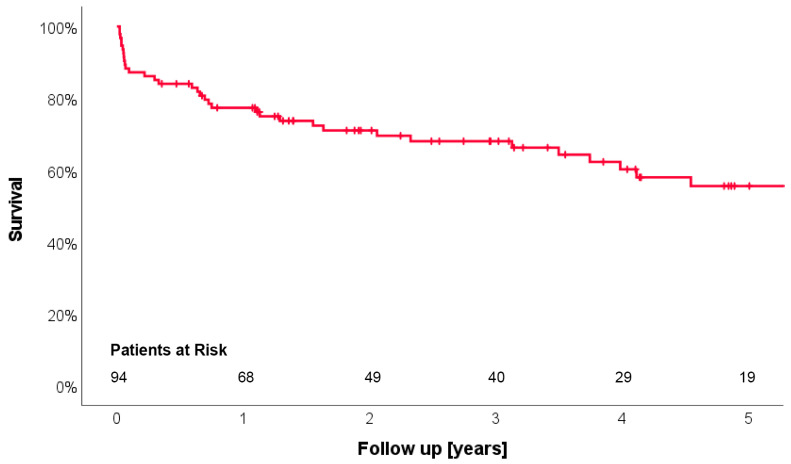
Kaplan–Meier curve for survival of the patients who underwent LVAD implantation with concomitant tricuspid valve repair. Survival at 1, 3 and 5 years was 77.4%, 68.1% and 55.6%.

**Figure 2:**
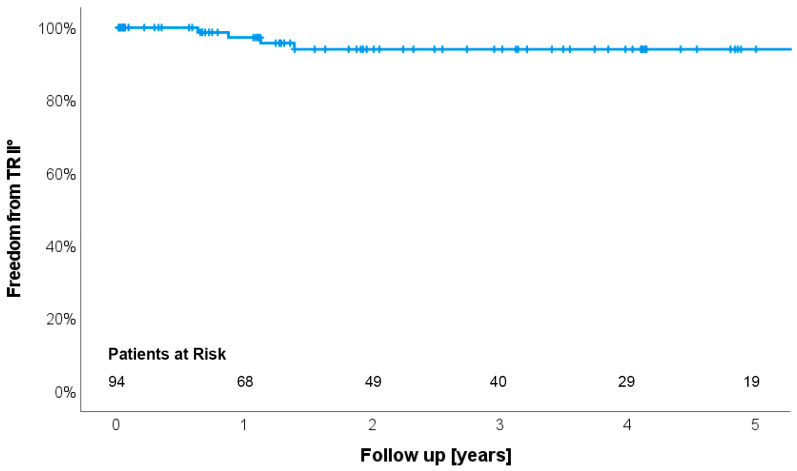
Kaplan–Meier curve for freedom from moderate or severe TR during follow-up. Freedom from moderate TR was 94.0% at five years.

**Figure 3:**
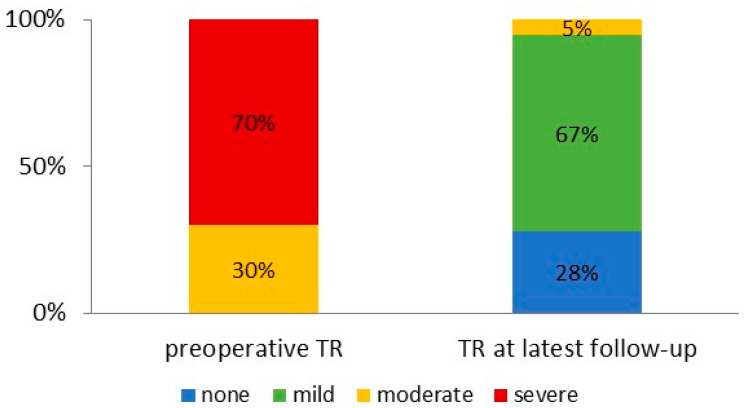
Distribution of tricuspid regurgitation rates from echocardiographic examinations before surgery or during follow-up.

Figure 1 shows the Kaplan–Meier curve for survival. It was 77.4%, 68.1% and 55.6% at 1, 3 and 5 years, respectively. Heart transplantation was performed in 12 patients (12.8%). During the entire follow-up period, 36 patients died. Table 3 shows clinical and echocardiographic parameters during follow-up. Mean follow-up duration was 2.8 years with a minimum of 7 days and a maximum of 10.6 years. Adverse events were pump thrombosis in two patients (2.1%), stroke in eight (8.5%) and gastro-intestinal bleeding in nine (9.6%). Clinical evidence for right heart failure was present in only two patients (2.0%), characterized by leg edema, dyspnea and LVAD low-flow alarms leading to rehospitalization. It was successfully treated conservatively (diuretics) and by pump speed adjustment. The individual latest echocardiographic follow-up showed no/trace TR in 26 (27.7%) patients, mild in 63 (67.0%) and moderate TR in 5 (5.3%). None of the patients presented with severe TR. Figure 2 shows the corresponding freedom from moderate or severe TR during follow-up with an actuarial freedom from moderate TR of 94.0% at five years. Figure 3 shows the distribution of TR degrees from echocardiographic examinations before surgery and during follow-up. Moderate TR was seen in five patients (5.3%) only. Not a single patient showed severe TR during follow-up (Table 3).

**Figure 4:**
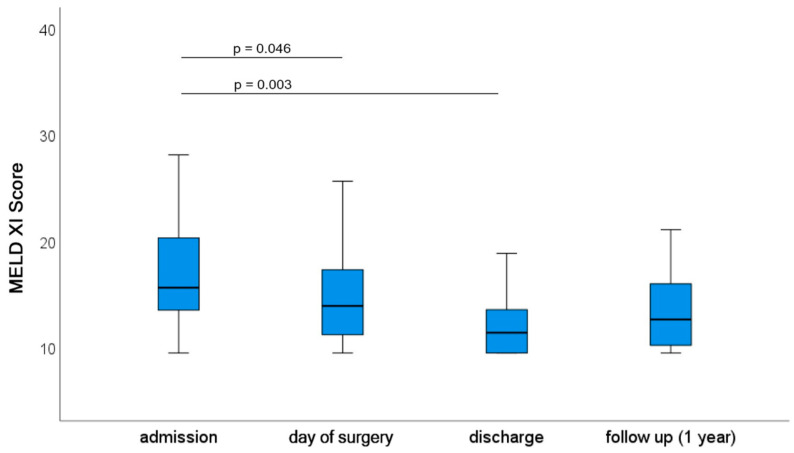
Distribution of MELD Xi score before surgery and during follow-up.

Figure 4 shows the MELD Xi score as a surrogate marker for the presence of liver dysfunction. The highest score was found on admission (17.3 ± 6.1) which was lowered during the preoperative recompensation phase (optimized heart failure medication, levosimendan, inotropic support; MELD Xi score: 15.3 ± 5.7; *p* < 0.05). The lowest score values were seen at discharge (12.9 ± 5.2; *p* < 0.05) and, at 1-year follow-up, MELD Xi remained relatively stable (14.1 ± 5.6).

## 5. Discussion

We demonstrate in this analysis that, contrary to expectations, concomitant tricuspid valve repair during LVAD implantation may result in excellent repair durability, which appears to be associated with low risk for right heart failure.

There is a current debate in the LVAD community regarding the value of concomitant tricuspid valve repair. It is a common notion that TR may not have to be addressed during LVAD implantation, because the natural course of the disease might not be predictable [23]. In addition, there appear to be high failure rates during follow-up and questionable clinical benefit. For instance, Barac et al. analyzed a series of 156 patients receiving additional tricuspid valve repair during LVAD implantation. The authors described recurrence of moderate to severe TR in 38% of patients and right heart failure in 36%, suggesting an independent association between repair and right heart failures [18]. In contrast, other investigators stress the importance of TVR by demonstrating early reverse remodeling of the right ventricle with a significant reduction in right ventricular end-diastolic area [24]. In addition, the ADVANCE investigators support this conclusion in a bridge to transplant patient population. Patients with preoperatively severe TR not receiving tricuspid valve repair showed an increased rate of right heart failure compared to those that received tricuspid annuloplasty [25]. Both of these findings are consistent with our results. We show excellent stability of our tricuspid valve repair, which was associated with a very low rate of right heart failures and other postoperative adverse events (dialysis, thromboembolic events, stroke, arrythmia, device failure). There were only two patients during follow-up presenting with clinically relevant right heart failure, as evident by edema, dyspnea and low-flow alarms. Two other patients experienced right heart failure during surgery, which was associated with their highly critical preoperative status (INTERMACS 1, preoperative ECLS). Thus, our findings would argue for a true value of TVR in LVAD patients with indication for TVR according to valve guidelines, which includes not only those with severe TR but also those with moderate TR and evidence for annular dilatation. In any case, the connection to a very low rate of right heart failure during follow-up is striking. While we cannot establish a direct and possibly causal relationship between TVR and right heart failure, we believe that our postoperative and follow-up management of the patients does not differ much from other centers to explain the remarkable difference.

Another strong argument for concomitant TVR during LVAD implantation is the fact that the long-term benefits come without increasing intraoperative risk. Han et al. showed that there was no difference in operative mortality or short- and long-term adverse events in their study cohort comparing LVAD patients with or without concomitant TVR [26]. As transcatheter treatment options for TVR are under investigation and redo surgery for the tricuspid valve carries higher risk, it appears reasonable to us to perform concomitant TVR more liberally at the time of LVAD implantation. In our patient cohort, indication for concomitant TVR was principally considered based on current ESC/EACTS guidelines for the management of valvular heart disease more generously than currently suggested for LVAD patients [27]. Consistent with others [26], we did not see an increase in perioperative adverse events (i.e., re-exploration for bleeding, RHF, stroke; data not shown) in our patient population.

Although the current literature shows superior durability for TVR, when performed with semirigid ring annuloplasty and less so for bands [28], our results demonstrate excellent TVR durability with band annuloplasty in all cases. We did not address the sizes of the individual bands in detail because we used the Simplici-T system, which is a 10 cm band and is tailored to the individual patient. The other band systems use the classic sizing nomenclature starting from size 26 to 34. Although there were some variations in sizes and lengths selected, we generally oriented ourselves on the length of a 28 Cosgrove band (which is 63 mm) and the vast majority of patients will therefore have received bands of lengths between 63 and 73 mm. Based on the low recurrence rate (only five patients had moderately recurrent TR), we believe that the length of the band is not the most critical factor, but the proper anchoring of the band along the annulus from the antero-septal commissure until the mid-portion of the septal annulus. TVR failure has been suggested to correlate with RHF in the five patients presenting with recurrent TR. It is possible that this relationship requires the presence of severe TR. However, two of the patients underwent heart transplantation within 2 years, one died on postoperative day 230 due to fulminant cerebral bleeding and the remaining two are alive and in a stable clinical condition after 2.9 and 6.2 years.

In general, our findings of competent and durable tricuspid valve repairs in this patient population associated with very low rates of right heart failure argue for a liberal performance of restrictive band annuloplasty. Our results support our understanding that surgical attention to valve regurgitation generating a competent and durable outcome has the potential to improve long-term results of patients with or without LVADs.

## 6. Limitations

The study is limited by its retrospective design and its limited patient number. The data describe a group experience without a comparative cohort. Furthermore, degree of TR might be influenced by each patient’s volume load and hemodynamic status. However, all patients were treated according to unified standard operative procedures including serial preoperative assessment (i.e., echocardiography). Nevertheless, our data demonstrate good and durable tricuspid valve repair results and low incidence of right heart failure in a patient cohort with severely impaired RV function.

## 7. Conclusions

We conclude that, contrary to expectations, concomitant tricuspid valve repair during LVAD implantation may result in excellent repair durability, which appears to be associated with low risk for right heart failure.

## Figures and Tables

**Table 1:** Preoperative Patient Characteristics.

		All Patients
		(n = 94)
Age [years]		59.8 ± 11.4
Male [%]		84 (89.4)
BMI [kg/m^2^]		26.9 ± 4.8
PM/ICD		56 (59.6)
COPD		10 (10.6)
Diabetes, insulin dependent		28 (39.8)
ICM/DCM		37 (39.4)/56 (59.6)
DT/BTT/BTD		40 (42.6)/31 (33)/23 (24.5)
INTERMACS level	1	15 (16.0)
	2	20 (21.3)
	3	30 (31.9)
	4	29 (30.9)
Preoperative MCS		12 (12.8)
*Echocardiographic data*		
Degree of TR	Moderate	28 (29.8)
	Severe	66 (70.2)
	Severe RV dysfunction	61 (64.9)
TAPSE [mm]		12.6 ± 3.8
*Right heart catheterization:*		
CVP [mmHg]		14.1 ± 6.8
Mean PAP [mmHg]		37.2 ± 13.2
PCWP [mmHg]		26.7 ± 8.7
Cardiac index [L/min/m^2^]		1.9 ± 0.6
PVR		235.2 ± 170.4
CVP/PCWP [mmHg]		0.53 ± 0.3
PAPi		1.9 ± 1.1
*EUROMACS right heart failure score*		
0–2		15 (15.9)
>2–4		19 (20.2)
>4		60 (63.8)

Continuous data are shown as mean ± standard deviation, categorical data are presented as crude numbers (% of total). BMI: body mass index, PM: pacemaker, ICD: implantable cardioverter defibrillator, COPD: chronic obstructive pulmonary disease, ICM: ischemic cardiomyopathy, DCM: dilative cardiomyopathy, DT: destination therapy, BTT: bridge to transplant, BTD: bridge to decision, INTERMACS: Interagency Registry for Mechanically Assisted Circulatory Support, MCS: mechanical circulatory support, TR: tricuspid valve regurgitation, RV: right ventricle: TAPSE: tricuspid annular plane systolic excursion, CVP: central venous pressure, PAP: pulmonary artery pressure, PCWP: pulmonary capillary wedge pressure, PVR: pulmonary vascular resistance, TPG: transpulmonary pressure gradient, PAPi: pulmonary artery pulsatility index.

**Table 2:** Operative Patient Data.

	All Patients
	(n = 94)
Procedural duration [hours]	3.8 ± 1.1
CPB time [min]	110.7 ± 30.5
Redo surgery	19 (20.2)
HeartMate II (Abbott)	14 (14.9)
HeartMate 3 (Abbott)	71 (75.3)
HVAD (Medtronic)	6 (6.4)
HeartAssist 5 (Micromed Cardiovascular)	3 (3.2)
TV annuloplasty ring type	
Medtronic Simplici-T	7 (50.0)
Cosgrove-Edwards	46 (48.9)
Medtronic Simulus	1 (1.1)
*Concomitant procedures (other than TVR)*	
Aortic valve replacement	14 (14.9)
ASD/PFO closure	18 (19.1)
CABG	3 (3.2)
*Postoperative adverse events*	
RVAD implantation	2 (2.1)
Renal insufficiency requiring dialysis	18 (19.1)
Pump thrombosis	none
Arrythmia	7 (7.4)
Visceral ischemia	2 (2.1)
Perioperative stroke	5 (5.3)

Continuous data are shown as mean ± standard deviation, categorical data are presented as crude numbers (% of total). CPB: cardiopulmonary bypass, TV: tricuspid valve, TVR: tricuspid valve repair, ASD: atrial septal defect, PFO: patent foramen ovale, CABG: coronary artery bypass graft, RVAD: right ventricular assist device.

**Table 3:** Clinical and Echocardiographic Parameters during Follow-up.

		All Patients
		(n = 94)
Follow-up time [years]		2.8 ± 2.3 (7 days to 10.6 years)
Pump thrombosis		2 (2.1)
Stroke		8 (8.5)
GI bleeding		9 (9.6)
Rehospitalization due to RHF		2 (2.0)
Heart transplantation		12 (12.8)
*Echocardiographic data*		
Degree of TR	None/trace	26 (27.7)
	Mild	63 (67.0)
	Moderate	5 (5.3)
	Severe	none

Continuous data are shown as mean ± standard deviation, categorical data are presented as crude numbers (% of total). GI: gastro-intestinal, RHF: right heart failure, TR: tricuspid valve.

## Data Availability

The underlying data of this article cannot be shared publicly as this has not been approved by the patients.

## Abbreviations

CO	cardiac output
CPB	cardiopulmonary bypass
CT	computed tomography
GI	gastro-intestinal
LVAD	left ventricular assist device
MCS	mechanical circulatory support
MELD	Model of End Stage Liver Disease
RHF	right heart failure
RVAD	right ventricular assist device
TR	tricuspid regurgitation
TVR	tricuspid valve repair
